# Pantethine Restores Epidermal Homeostasis and Modulates Vesicle‐Associated Proteins in Psoriasis

**DOI:** 10.1111/exd.70381

**Published:** 2026-10-30

**Authors:** Passanesh Sukphopetch, Kamonpan Fongsodsri, Pornanong Aramwit, Onrapak Reamtong, Tipparat Thiangtrongjit, Tapanee Kanjanapruthipong, Watcharamat Muangkaew, Sumate Ampawong

**Affiliations:** ^1^ Department of Microbiology and Immunology, Faculty of Tropical Medicine Mahidol University Bangkok Thailand; ^2^ Department of Tropical Pathology, Faculty of Tropical Medicine Mahidol University Bangkok Thailand; ^3^ Department of Pharmacy Practice, Faculty of Pharmaceutical Sciences and Center of Excellence in Bioactive Resources for Innovative Clinical Applications Chulalongkorn University Bangkok Thailand; ^4^ The Academy of Science The Royal Society of Thailand Bangkok Thailand; ^5^ Faculty of Pharmacy Silpakorn University Nakhon Pathom Thailand; ^6^ Department of Molecular Tropical Medicine and Genetics, Faculty of Tropical Medicine Mahidol University Bangkok Thailand; ^7^ Mahidol Oxford Tropical Medicine Research Unit, Faculty of Tropical Medicine Mahidol University Bangkok Thailand

**Keywords:** epidermal homeostasis, exosomes, immunoregulatory, pantethine, psoriasis, SLIT/ROBO pathway

## Abstract

Psoriasis is a chronic inflammatory skin disease characterized by disrupted epidermal homeostasis driven by interconnected immuno‐inflammatory, metabolic, and redox signalling networks. Vesicle‐associated intercellular communication has emerged as an important contributor to inflammatory amplification and tissue remodelling within the epidermal microenvironment. Pantethine, a stable vitamin B5 derivative with lipid‐modulatory and anti‐inflammatory properties, has been implicated in regulation of cellular stress responses and membrane‐associated signalling pathways, suggesting potential therapeutic relevance in psoriasis. Here, we investigated the effects of pantethine using a full‐thickness 3D human psoriatic skin model following keratinocyte safety assessment. Integrated histopathological, immunohistochemical, quantitative RT‐PCR, and proteomic analyses were employed to interrogate inflammatory, oxidative, proliferative, apoptotic, differentiation‐associated and vesicle‐related responses. Pantethine significantly reduced epidermal thickening and normalized multiple psoriasis‐associated molecular signatures. Proteomic profiling revealed coordinated modulation of vesicle‐associated, cytoskeletal and extracellular matrix‐related proteins, accompanied by reduced expression of exosome‐associated markers CD63 and CD9. Pantethine suppressed pro‐inflammatory signalling involving IL‐1β, TNF‐α, IL‐17, *S100* family members, and hyperproliferative keratin‐associated pathways while reducing caspase‐3–associated apoptotic activity. Concurrently, pantethine enhanced antioxidative and immunoregulatory responses through activation of Nrf‐2 and TGF‐β, restored differentiation and barrier‐related markers including *filaggrin*, *caspase‐14*, and *involucrin*, and reduced hypoxia‐ and tissue‐remodelling–associated mediators including HIF‐1α, VEGF, COL6A3, and COL1A2. Network‐based proteomic analyses further identified computationally inferred associations with SLIT/ROBO‐related pathways, although these associations require further functional validation. Collectively, these findings support pantethine as a promising candidate that modulates vesicle‐associated proteins together with inflammatory and tissue‐remodelling responses involved in epidermal homeostasis.

## Graphical abstract

Pantethine attenuates psoriasis‐like epidermal pathology in a full‐thickness 3D human skin model by modulating inflammatory, oxidative‐stress, differentiation and tissue‐remodelling responses, alongside changes in vesicle‐associated proteins. These findings support restoration of epidermal homeostasis, although direct effects on extracellular vesicles require further validation.

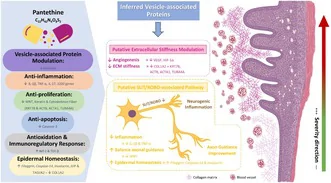

## 1 Introduction

Psoriasis is a chronic inflammatory skin disorder characterized by keratinocyte hyperproliferation, aberrant differentiation, immune cell infiltration and persistent inflammation. Rather than arising from a single dominant pathway, disease pathogenesis reflects dysregulated integration of immune‐mediated and intracellular signalling networks within the epidermal–immune axis, ultimately disrupting epidermal homeostasis. A self‐amplifying pro‐inflammatory microenvironment, driven by key cytokines and chemokines—including tumour necrosis factor‐alpha (TNF‐α), interleukin (IL)‐1β, IL‐6, IL‐8, IL‐21, IL‐22, and C‐C motif chemokine ligand 20 (CCL‐20)—facilitates recruitment of C‐C chemokine receptor 6–positive (CCR6^+^) T helper 17 (Th17) cells and promotes IL‐17/IL‐22–mediated epidermal activation [1, 2]. In parallel, dysregulated expression of innate immune effectors such as β‐defensins and apoptosis‐associated caspase‐3 exacerbates keratinocyte turnover and barrier dysfunction [3, 4, 5], while protective homeostatic signalling pathways mediated by nuclear factor erythroid 2–related factor 2 (Nrf‐2) and transforming growth factor‐beta (TGF‐β) are frequently attenuated [6].

Psoriatic disease progression is driven by interconnected signalling networks rather than isolated linear cascades. Nuclear factor kappa‐light‐chain‐enhancer of activated B cells (NF‐κB; p65 subunit) sustains inflammation and keratinocyte proliferation through TNF‐α‐ and IL‐32–dependent signalling [7, 8], while Janus kinase/signal transducers and activators of transcription (JAK–STAT; STAT2/3) pathways mediate cytokine‐driven immune amplification [9]. Mitogen‐activated protein kinase (MAPK) signalling, including p38 MAPK, extracellular signal‐regulated kinases 1/2 (ERK1/2), and c‐Jun N‐terminal kinase (JNK), further contributes to epidermal hyperplasia and inflammatory signalling [10]. In addition, wingless‐related integration site (WNT) and mechanistic target of rapamycin (mTOR) pathways regulate cytokine production, autophagy suppression and epidermal imbalance [11, 12]. Although biologics and small‐molecule inhibitors targeting these nodes have improved clinical outcomes, their limited durability and incomplete disease control underscore the challenge of modulating complex upstream and downstream signalling integration. Therapeutic targeting of regulators such as myeloid differentiation primary response 88 (MYD88) and mucosa‐associated lymphoid tissue lymphoma translocation protein 1 (MALT1), as well as downstream effectors including JAKs and mTOR, has highlighted both the promise and the limitations of current interventions, with incomplete responses, adverse effects and disease relapse remaining common [9, 13, 14]. Collectively, these observations emphasize the need for complementary strategies capable of reprogramming pathogenic signalling networks while restoring epidermal homeostasis.

Beyond canonical cytokine signalling, extracellular vesicles (EVs) have emerged as integral components of psoriatic disease biology. Keratinocyte‐derived EVs (kEVs) promote neutrophil activation and epidermal hyperplasia, whereas circulating EV‐associated microRNAs correlate with disease activity and psoriatic arthritis [15, 16]. Conversely, small EVs (sEVs) derived from mesenchymal stem cells attenuate inflammation by suppressing IL‐17A, interferon‐gamma (IFN‐γ), IL‐6 and TNF‐α, resulting in reduced epidermal thickening and erythema [17, 18]. Fibroblast‐derived EVs (fEVs) further contribute to skin regeneration through MAPK‐dependent keratinocyte proliferation and migration [19, 20]. These findings position EVs as dynamic mediators within broader signalling ecosystems rather than isolated pathogenic drivers. Nevertheless, therapeutic strategies directly targeting exosome biogenesis or function in psoriasis remain limited. While inhibitors such as GW4869 and simvastatin suppress exosome biogenesis and exhibit anti‐psoriatic effects [17, 21, 22], other agents (e.g., glibenclamide and indomethacin) have been reported to exacerbate disease severity [23], highlighting the context‐dependent roles of vesicle‐associated signalling.

Pantethine, a stable derivative of vitamin B5 and a precursor of coenzyme A, participates in lipid metabolism, fatty acid oxidation and cellular detoxification through more than 70 enzymatic reactions [24]. Clinically, pantethine has been shown to improve lipid profiles, support vascular function and reduce oxidative stress. Emerging evidence suggests that pantethine may influence vesicle dynamics and lipid‐dependent signalling processes, potentially intersecting with sphingomyelinase‐regulated pathways involved in intercellular communication [25]. Although its role in psoriasis has not been previously defined, its favourable safety profile and pleiotropic biochemical properties position pantethine as a candidate modulator of vesicle‐associated pathogenic signalling.

In this study, we investigated whether pantethine can modulate vesicle‐associated intercellular communication and reprogram pathogenic signalling networks governing epidermal homeostasis using a full‐thickness 3D human psoriatic skin model. Cytotoxicity was first evaluated in HaCaT keratinocytes, followed by histopathological, immunohistochemical, quantitative real‐time PCR, and proteomic analyses to delineate alterations in inflammatory, oxidative stress–responsive, proliferative and apoptotic pathways. By integrating vesicle‐associated signalling with downstream molecular and phenotypic outputs, this work provides mechanistic insight into how pantethine reshapes pathogenic communication circuits within the epidermal–immune interface.

## 2 Materials and Methods

### 2.1 Reagents, Chemicals and Cells

Details of reagents, materials, and suppliers used in this study are summarized in Table S1.

### 2.2 Cytotoxicity and Dose Selection of Pantethine

To evaluate the cytotoxicity and determine suitable concentrations of pantethine for subsequent experiments, HaCaT keratinocytes were seeded at 7000 cells/well in 96‐well plates and incubated overnight at 37°C with 5% CO_2_. Following phosphate‐buffered saline (PBS) washing, cells were treated with at least five concentrations of pantethine for 24 h. Pantethine (300 mg per softgel; NOW Foods, USA; catalogue No. 0487B v7) was obtained as a commercial softgel formulation. For preparation of the stock solution, the pantethine active ingredient was separated from the capsule shell and excipients and dissolved in 10 mL of absolute dimethyl sulfoxide (DMSO) to obtain a nominal concentration of 30 mg/mL. The solution was vortexed until homogeneous and passed through a 0.22‐μm syringe filter. The resulting stock solution was serially diluted with the appropriate culture medium to prepare final pantethine concentrations of 1 ng/mL, 10 ng/mL, 100 ng/mL, 1000 ng/mL, 20 μg/mL, 40 μg/mL, 80 μg/mL, 100 μg/mL and 500 μg/mL for the cytotoxicity assay. Cell viability was then assessed using the MTT assay, as previously described [26]. Concentration ranges for pantethine treatment were selected based on cytotoxicity profiles derived from MTT assays. Based on the MTT results, three doses (100, 500 and 1000 ng/mL) were selected to reflect a gradient of cellular responses, spanning from minimal to moderate reduction in viability. This tiered approach allowed for the exploration of both subtherapeutic and pharmacologically active levels, facilitating a more nuanced interpretation of pantethine's biological impact.

### 2.3 Pantethine Treatment Protocol

To evaluate the potential anti‐psoriatic effects of pantethine, three concentrations (100, 500 and 1000 ng/mL) were tested alongside a reference treatment consisting of diluted Xamiol gel (LEO Pharma, Denmark), a fixed‐combination formulation containing calcipotriol (50 μg/g) and betamethasone dipropionate (0.5 mg/g). The gel was diluted to provide a final calcipotriol concentration of 1 μg/g before application to the skin equivalents. Untreated psoriatic skin equivalents and untreated normal skin equivalents served as disease and normal controls, respectively. A commercially available full‐thickness reconstituted human psoriatic skin model (SOR‐300‐FT) and its matched non‐psoriatic counterpart (SOR‐300‐FT‐CTR) were utilized. Each experimental condition was conducted in triplicate.

The 3D skin equivalents, supported on a porous hanging membrane within 24‐well culture plates, were maintained in accordance with the manufacturer's recommendations and following optimized procedures established in our previous studies. Prior to treatment initiation, skin equivalents were equilibrated under standard culture conditions (37°C, 5% CO_2_) for 16–18 h to ensure barrier integrity and tissue viability. During the treatment phase, pantethine, Xamiol or vehicle control were applied topically (50 μL) to the epidermal surface on alternate days, and the culture medium specific to each model type (SOR‐300‐FT‐MM for psoriatic and SOR‐300‐FT‐CTR‐MM for normal skin) was replenished every other day.

After a 7‐day treatment period, each tissue construct was divided into three portions: one was fixed in 10% neutral‐buffered formalin (NBF) for immunohistopathological analysis, another was preserved in RNAlater (Thermo Scientific, USA) at −20°C for subsequent gene expression analysis, and the remaining portion was frozen at −80°C for proteomic studies.

### 2.4 Histopathological Study

To assess epidermal thickness, histopathological analyses of the artificial skin equivalents were performed to evaluate thickening from acanthosis, rete ridge elongation and hyperkeratinization [11, 26, 27, 28]. Tissue samples were fixed in 10% NBF for 24 h and subsequently processed using conventional histological techniques, including graded ethanol dehydration, xylene and paraffin infiltration, and paraffin embedding. Paraffin blocks were sectioned at 5 μm thickness, and sections were stained with haematoxylin and eosin (H&E). Morphometric evaluation was performed using a light microscope (BX51, Olympus, Japan) equipped with a digital imaging system (DP70, Olympus, Japan). Epidermal thickness, defined as the perpendicular distance spanning from the basal layer to the stratum corneum, was quantified using ImageJ software (version 1.5 J8, NIH), with measurements obtained from five randomly selected fields per sample at 400× magnification.

### 2.5 Immunohistochemistry (IHC) Study

To investigate the effects of pantethine on inflammation and hypersensitivity mediated through innate, adaptive and auto‐amplification loop immune responses, as well as on keratinocyte proliferation, antioxidant capacity, apoptosis, hypoxia‐inducible inflammatory mediators and proteomic validation markers (e.g., exosomal markers and extracellular matrix stiffness factors), immunohistochemical analyses were conducted following protocols established in our previous studies [11, 26, 27, 28].

Artificial skin sections from each experimental group were subjected to a rigorous immunohistochemical workflow to characterize protein expression within the epidermal compartment. Sections were first deparaffinized in xylene and then exposed to heat‐induced antigen retrieval in citrate buffer (pH 6.0) to unmask epitopes. Endogenous peroxidase activity was effectively quenched using 1% hydrogen peroxide in methanol and non‐specific binding sites were blocked with a serum‐free protein block solution. Sections were subsequently incubated with primary antibodies at 1:100 or 1:200 dilutions in PBS containing 1% normal goat serum, followed by detection using an HRP‐labelled polymer system (EnVision, Dako). Visualization of antibody binding was achieved with DAB chromogen, and counterstaining with haematoxylin was performed prior to permanent mounting with DPX.

Protein expression was semi‐quantitatively evaluated using the H‐score, which integrates both the proportion and intensity of positively stained areas on a scale from 0 (no expression) to 300 (strong expression). For each sample, at least five randomly selected fields were captured at 400× magnification (640 × 480 pixels) using a light microscope. Images were converted to 8‐bit grayscale in ImageJ software (version 1.5 J8, NIH), and positively stained regions were identified as black pixels. The area fraction of these regions within the epidermis was calculated to determine the percentage of protein expression. In parallel, staining intensity was graded on a four‐level scale: 0 = no staining, 1 = low, 2 = medium and 3 = high. H‐scores were then calculated for each sample to enable robust comparisons across experimental groups.

### 2.6 Determination of Genes Involving Psoriasis

Quantitative real‐time PCR (qRT‐PCR) was employed to evaluate the effects of pantethine on mRNA expression associated with keratinocyte turnover and epidermal integrity/homeostasis. Artificial skin samples treated with the respective test substances were processed using the RNeasy Mini Kit (Qiagen, Canada). Briefly, tissues were homogenized in 600 μL of lysis buffer, and the lysates were centrifuged to remove debris. The resulting supernatants were loaded onto spin columns, where RNA was bound, sequentially washed and subsequently eluted in 35 μL of RNase‐free water. RNA yield and purity were determined using a NanoDrop 2000/2000c spectrophotometer (Thermo Scientific, USA).

Complementary DNA synthesis and amplification were carried out with iTaq Universal SYBR Green Supermix (Bio‐Rad, USA) on a CFX96 Touch Real‐time PCR system (Bio‐Rad, USA). The primer sequences employed in this study are listed in Table S2. Relative gene expression was quantified using the 2^−∆∆Ct^ method, and results were compared across experimental groups.

### 2.7 Proteomic Workflow for Assessing Pantethine Effects

To explore the mechanistic therapeutic potential of pantethine in psoriasis, we performed a comparative proteomic analysis using psoriatic 3D human skin models treated with 500 ng/mL pantethine—a concentration identified through the aforementioned assays as providing the most effective therapeutic response—or left untreated.

For protein isolation, tissues from treated and control groups were homogenized in lysis buffer (1% SDS, 1% Triton X‐100, 0.5% NaCl) under ultrasonication on ice, followed by centrifugation at 10 000×*g* for 10 min at 4°C. The supernatants were collected, and protein concentrations were determined by Bradford assay (Bio‐Rad, USA).

Subsequently, proteins were precipitated with ice‐cold acetone (1:5, v/v), pelleted, and resuspended in 0.25% RapidGest SF (Waters, USA) dissolved in 15 mM ammonium bicarbonate (Sigma‐Aldrich, USA). Aliquots containing 60 μg protein from each group underwent gel‐free enzymatic digestion. Reduction was performed with 5 mM DTT at 72°C for 1 h, followed by alkylation with IAA for 30 min in the dark. After desalting with Zeba Spin Columns (Thermo Scientific, USA), tryptic digestion (Promega, USA) was carried out at 37°C for 3 h. The resulting peptides were dried and reconstituted in 0.1% formic acid prior to LC–MS/MS. All experiments were performed in triplicate using independent biological replicates.

Mass spectrometry analysis was carried out in positive ion mode on an HF‐X Hybrid Quadrupole‐Orbitrap system coupled with EASY‐nLC1000 nano‐LC and a C18 column. Separation was achieved using a binary gradient of 0.1% formic acid (mobile phase A) and 90% acetonitrile with 0.1% formic acid (mobile phase B).

Protein intensity values generated by MaxQuant version 2.4.2.0 were subsequently analysed using Perseus software (version 2.1.5). Raw spectra (.RAW files) were searched against the UniProt *Homo sapiens* database (https://www.uniprot.org/) using an MS1 tolerance of 20 ppm, an MS/MS tolerance of 0.05 Da, trypsin specificity, carbamidomethylation of cysteine as a fixed modification and methionine oxidation as a variable modification. The false discovery rate (FDR) was controlled at 1% at both peptide and protein levels. Label‐free quantification (LFQ) intensity values were log_2_‐transformed and normalized prior to statistical analysis. Proteins identified only by site, reverse database hits and potential contaminants were excluded. Proteins with missing values were filtered according to the default Perseus workflow, and the remaining missing values were imputed from a normal distribution to simulate signals below the detection limit. Differential protein abundance between untreated and pantethine‐treated groups was assessed using a two‐sample Student's *t*‐test. Proteins with *p* < 0.05 together with fold‐change thresholds of ≥ 2.0 for upregulated proteins and ≤ 0.5 for downregulated proteins were considered differentially expressed. Identified proteins were functionally annotated using UniProt and PubMed, and protein–protein interaction networks were generated using STRING version 12.0 (www.string‐db.org). The identified differentially expressed proteins were used as the input dataset, with the whole genome as the statistical background. Functional enrichment was assessed using the enrichment analysis implemented in these databases, and pathways with a false discovery rate (FDR) ≤ 0.05 were considered statistically significant. Enrichment scores and FDR values were used to evaluate the strength and statistical significance of pathway associations.

The mass spectrometry proteomics data have been deposited to the ProteomeXchange Consortium via the PRIDE partner repository with the dataset identifier PXD082665 (https://doi.org/10.6019/PXD082665) [29]. The dataset is currently under controlled access during peer review and will be made publicly accessible upon publication of the manuscript.

### 2.8 Statistical Analysis

Statistical analyses were performed using SPSS Statistics v18, and data are expressed as mean ± SEM. For datasets following normal distribution, independent *t*‐tests or one‐way ANOVA were applied, whereas non‐parametric alternatives (Mann–Whitney U or Kruskal–Wallis) were used for non‐normal data. Statistical significance was defined as *p* < 0.05.

## 3 Results

### 3.1 Determining the Safe and Effective Dose of Pantethine in Keratinocytes

Pantethine concentrations that maintained ≥ 80% viability in HaCaT keratinocytes were identified using an MTT assay (Figure 1A). Based on these results, concentrations of 100, 500 and 1000 ng/mL were selected for subsequent experiments.

**FIGURE 1:**
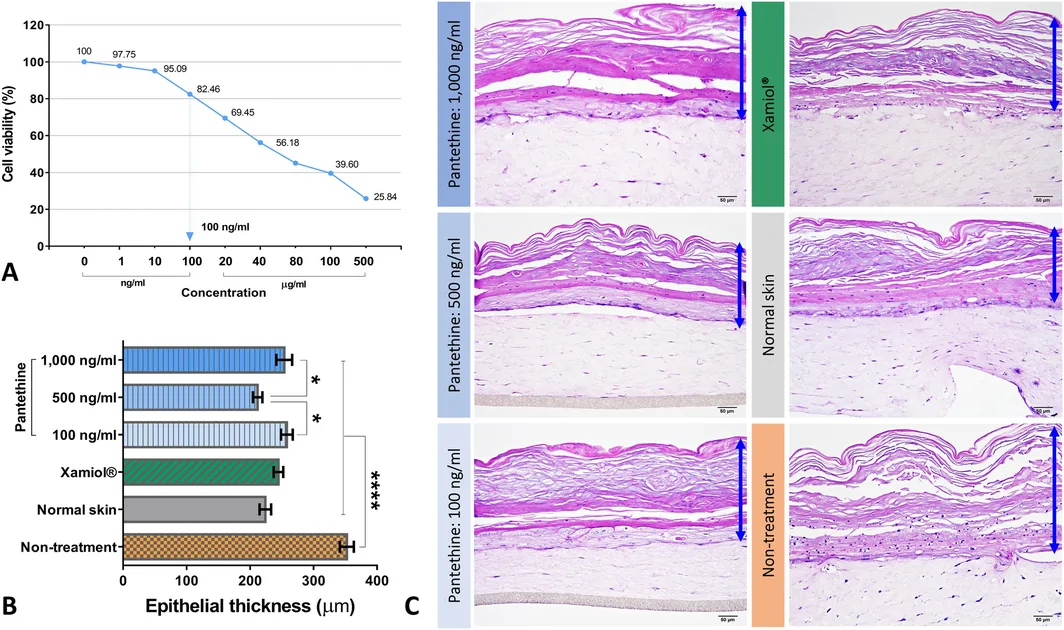
Assessment of cellular viability and epidermal thickening in psoriatic skin treated with various test compounds: (A) Cytotoxicity evaluation of pantethine at five different concentrations using the MTT assay in the HaCaT cell line. (B) Bar graph illustrating the extent of epidermal thickening following treatment with different compound groups. For histological analysis, three independent skin samples/sections were analysed per group, with five representative fields quantified per section (15 fields per group). Statistical analysis was performed using one‐way ANOVA (*p < 0.05, **p < 0.01, ***p < 0.001, ****p < 0.0001). (C) Histopathological micrographs depicting the severity of psoriasis in terms of epidermal thickening in treatment groups, including pantethine at concentrations of 1000, 500 and 100 ng/mL, the standard treatment group (Xamiol), normal skin and the untreated control group. Blue arrows denote epidermal thickening extending from the stratum basale to the stratum corneum.

### 3.2 Pantethine Ameliorates Psoriatic Epidermal Thickening

Histopathological analysis demonstrated that pantethine treatment at all tested concentrations significantly reduced epidermal thickness compared with untreated psoriatic controls (Figure 1B,C). The degree of reduction was comparable to that observed in the standard treatment and normal skin equivalents. Among the tested concentrations, pantethine at 500 ng/mL produced the most pronounced reduction in epidermal thickness. Although certain cytokine and regulatory markers exhibited concentration‐dependent variation, pantethine at 500 ng/mL demonstrated the most consistent overall improvement in epidermal architecture and regulatory marker balance and was therefore selected for downstream mechanistic analyses.

### 3.3 Immunomodulatory and Proteomics‐Validated Exosomal and Tissue‐Remodelling Effects of Pantethine

Immunohistochemical analyses were performed to quantify epidermal expression of psoriasis‐associated inflammatory, regulatory, exosomal and tissue‐remodelling markers using H‐score evaluation (Figures 2, 3, 4). Overall, pantethine treatment modulated multiple pathological features characteristic of psoriatic skin, with effects comparable to those observed in the standard treatment and normal skin equivalents.

**FIGURE 2:**
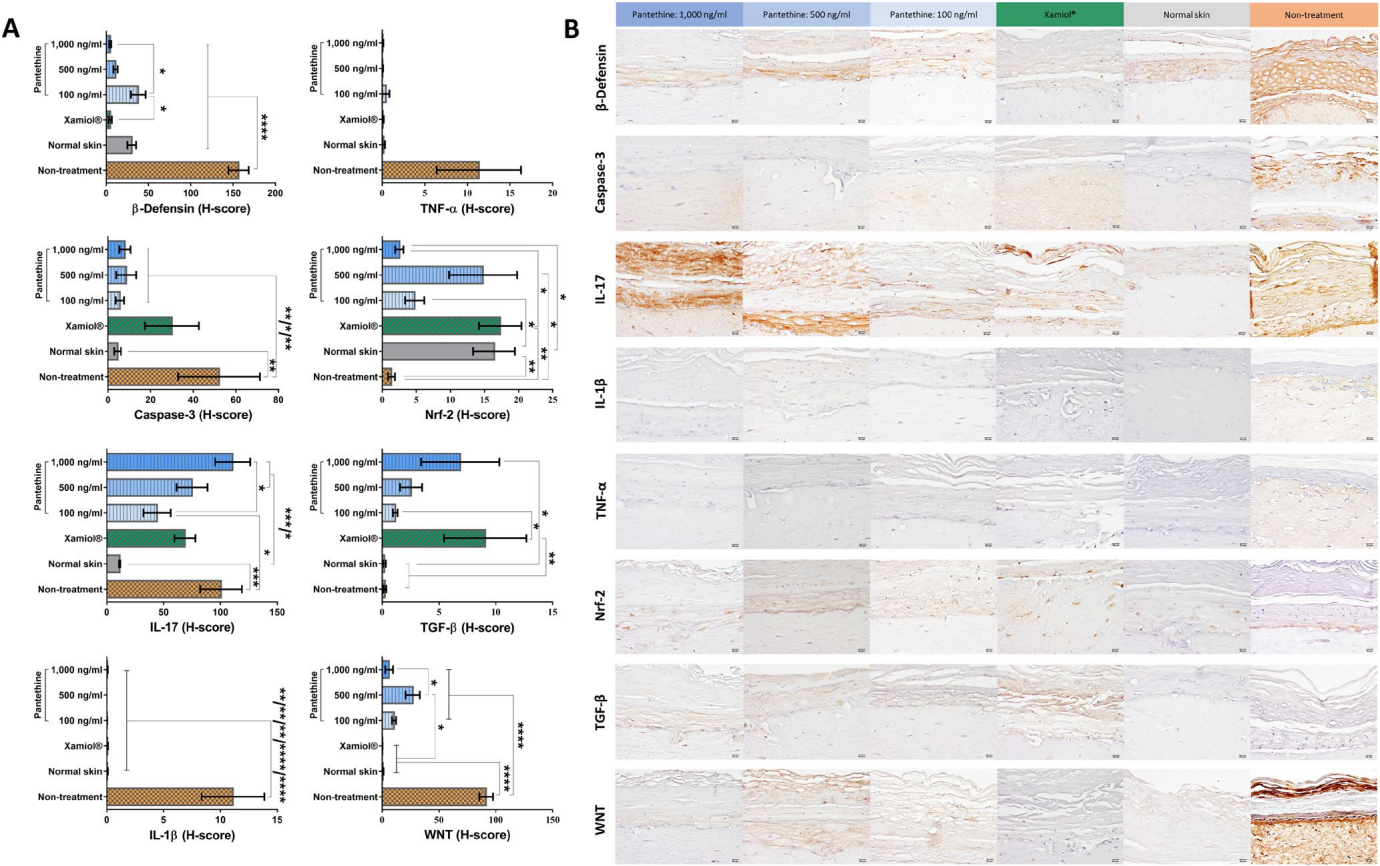
Quantification of critical immunological markers associated with psoriasis severity: Bar graphs illustrating the expression levels of β‐defensin, Caspase‐3, IL‐17, IL‐1β, TNF‐α, Nrf‐2, TGF‐β and WNT (A) in psoriatic skin equivalents following treatment with various test compounds. Three independent skin sections were analysed per group, with five representative fields quantified per section (15 fields per group). Statistical analysis was performed using one‐way ANOVA (*p < 0.05, **p < 0.01, ***p < 0.001, ****p < 0.0001). (B) Representative immunohistochemical micrographs demonstrating epidermal immunolocalization of each marker across the experimental groups. Positive immunoreactivity is identified by brown DAB chromogen deposition within epidermal area, including those treated with pantethine at concentrations of 1000, 500 and 100 ng/mL, the standard treatment group (Xamiol), normal skin and the untreated control group.

Pantethine significantly reduced the epidermal expression of key inflammatory and hyperproliferative markers, including β‐defensin, caspase‐3, IL‐1β, TNF‐α, IL‐17, WNT and KRT6A, across all tested concentrations compared with untreated psoriatic controls (Figure 2A). The magnitude of suppression was generally comparable to that observed in Xamiol‐treated and normal skin equivalents. Notably, IL‐17 expression was most effectively reduced at 100 ng/mL pantethine, reaching levels comparable to normal skin.

Markers associated with antioxidant and regulatory responses displayed concentration‐dependent modulation. Nrf‐2 expression was significantly increased at 500 ng/mL pantethine, reaching levels similar to normal skin and exceeding those observed at 100 and 1000 ng/mL (Figure 2A). In contrast, TGF‐β expression was most prominently upregulated at 1000 ng/mL pantethine and in the standard treatment group, relative to untreated controls.

In contrast, pantethine treatment did not significantly alter the expression of several pro‐inflammatory chemokines and cytokines, including CCL‐20, IL‐21, IL‐22, IL‐6 and IL‐8, at any tested concentration (Figure 3A).

**FIGURE 3:**
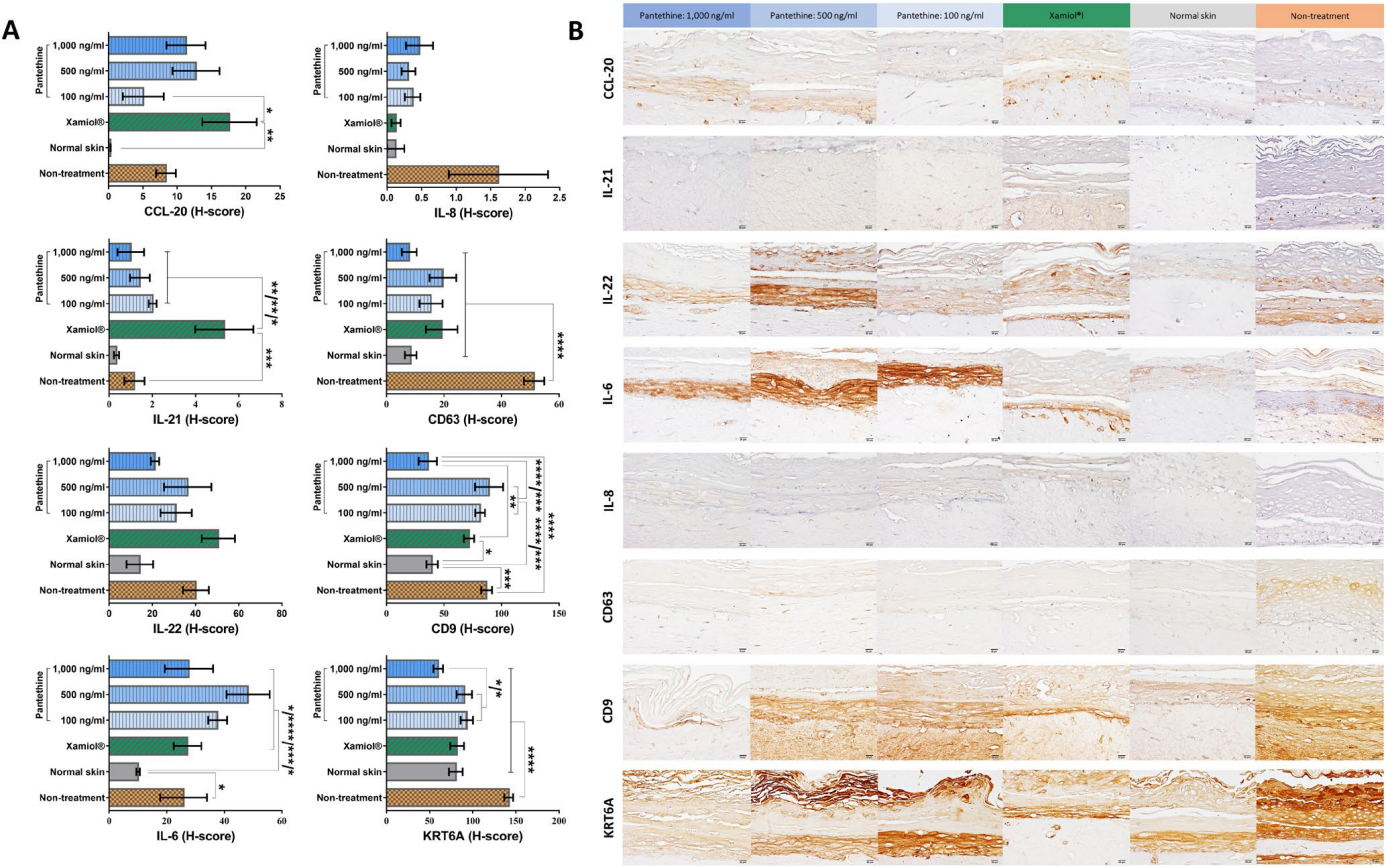
Quantification of additional immunological and exosome‐associated markers linked to psoriasis severity: Bar graphs illustrating the expression levels of CCL‐20, Il‐21, IL‐22, IL‐6, IL‐8, CD63, CD9 and KRT6A (A) in psoriatic skin equivalents following treatment with various test compounds. Three independent skin sections were analysed per group, with five representative fields quantified per section (15 fields per group). Statistical analysis was performed using one‐way ANOVA (*p < 0.05, **p < 0.01, ***p < 0.001, ****p < 0.0001). (B) Representative immunohistochemical micrographs demonstrating epidermal immunolocalization of each marker across the experimental groups. Positive immunoreactivity is identified by brown DAB chromogen deposition within epidermal area, including those treated with pantethine at concentrations of 1000, 500 and 100 ng/mL, the standard treatment group (Xamiol), normal skin and the untreated control group.

Proteomics‐guided validation of exosomal markers demonstrated a significant reduction in CD63 expression across all pantethine‐treated groups and the standard treatment group compared with untreated controls, with levels comparable to normal skin (Figure 3B). CD9 expression was significantly decreased only at the highest pantethine concentration (1000 ng/mL), whereas lower concentrations showed expression levels comparable to untreated controls. Additionally, immunohistochemical analysis revealed reduced epidermal expression of KRT6A following pantethine and standard treatments relative to untreated psoriatic skin.

Further validation of the proteomic findings, together with assessment of hypoxia‐inducible inflammatory mediators, demonstrated that epidermal expression of COL1A2, HIF‐1α, and VEGF was significantly reduced in all pantethine‐ and Xamiol‐treated groups compared with untreated controls (Figure 4A,B). COL1A2 and VEGF exhibited dose‐dependent decreases, with the lowest expression observed at 1000 ng/mL pantethine. At this concentration, COL1A2 expression was comparable to normal skin, whereas VEGF levels were reduced below those of normal skin. HIF‐1α expression was similarly suppressed across all treatment groups.

**FIGURE 4:**
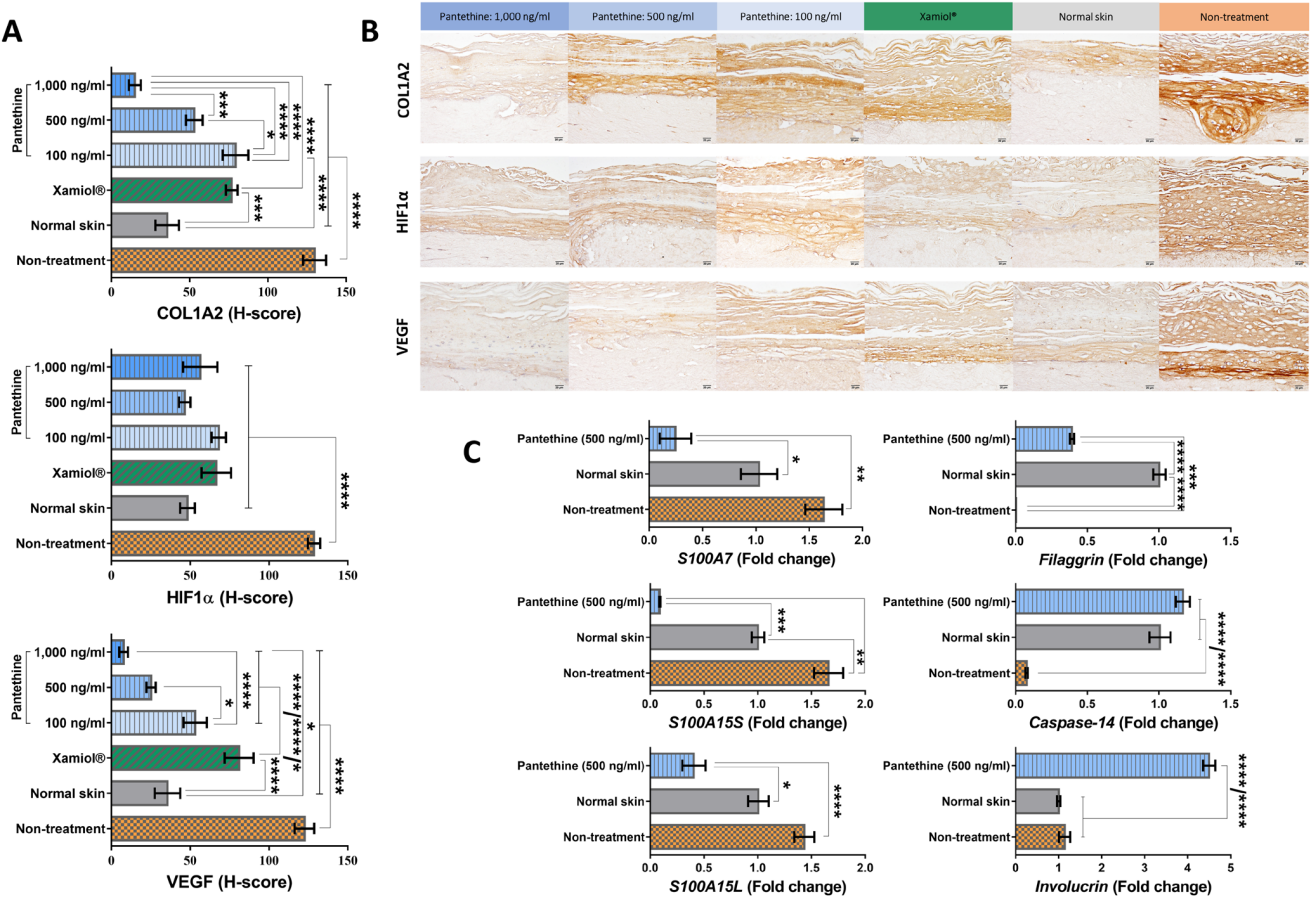
Validation of proteomic markers and treatment‐induced gene expression changes in psoriasis‐like skin equivalents: (A) Quantitative analysis of protein expression levels of COL1A2, HIF‐α and VEGF in psoriatic skin equivalents following treatment with various test compounds, as determined by proteomic validation. Three independent skin sections were analysed per group, with five representative fields quantified per section (15 fields per group). Data are presented as bar graphs. Statistical analysis was performed using one‐way ANOVA (*p < 0.05, **p < 0.01, ***p < 0.001, ****p < 0.0001). (B) Representative immunohistochemical micrographs showing epidermal immunolocalization of COL1A2, HIF‐1α and VEGF across experimental groups. Positive immunoreactivity is identified by brown DAB chromogen deposition within epidermal area, including pantethine‐treated samples (1000, 500 and 100 ng/mL), the standard treatment control (Xamiol), normal skin equivalents, and untreated psoriatic controls. For quantitative immunohistochemical analysis, three independent skin sections were analysed per group, with five representative fields quantified per section (15 fields per group). (C) Relative mRNA expression levels of psoriasis‐associated genes, including *S100A7*, *S100A15S*, *S100A15L*, *filaggrin*, *caspase‐14* and *involucrin*, in psoriasis‐like skin equivalents following the indicated treatments. Gene expression analyses were performed using three independent skin samples per group (*n* = 3).

### 3.4 Pantethine Regulates Psoriasis‐Associated Gene Expression

Quantitative real‐time PCR analysis revealed that pantethine treatment at 500 ng/mL significantly downregulated psoriasis‐associated genes, including *S100A7*, *S100A15S* and *S100A15L*, compared with untreated controls and normal skin equivalents (Figure 4C). In contrast, the expression of epidermal differentiation and barrier‐related genes, including *filaggrin*, *caspase‐14* and *involucrin*, was significantly upregulated relative to untreated controls, although *filaggrin* expression remained lower than that observed in normal skin.

### 3.5 Proteomic Insights Into Pantethine's Therapeutic Action

To elucidate molecular mechanisms associated with pantethine‐mediated attenuation of psoriasis‐like pathology, a comparative proteomic analysis was performed using psoriatic skin equivalents treated with pantethine at 500 ng/mL and untreated controls. A total of 365 proteins were identified, of which 74 exhibited statistically significant differential expression (*p* < 0.05). Applying fold‐change thresholds (≥ 2.0 for upregulation and ≤ 0.5 for downregulation), 22 proteins were upregulated and 12 proteins were downregulated following pantethine treatment (Tables 1 and 2).

**TABLE 1:** Upregulated proteins in pantethine (500 ng/mL)‐treated psoriatic skin equivalents compared to untreated controls.

Protein name	Protein accession	Fold change	Coverage (%) (≥ 10)	Protein score (≥ 25)
Keratinocyte differentiation‐associated protein	KRTDAP	3.830	16.2	16.071
Keratin, Type II cytoskeletal 6A	KRT6A	3.421	59	185.71
Transgelin‐2	TAGLN2	3.230	27.6	30.372
Vinculin	Vcl	3.205	2	6.3778
Zinc finger homeobox protein 3	ZFHX3	3.125	0.2	5.4881
Small ribosomal subunit protein uS13	RPS18	2.944	5.9	5.7141
6‐phosphogluconate dehydrogenase, decarboxylating	PGD	2.683	4.8	8.3714
Keratin, Type II cytoskeletal 79	KRT79	2.584	14.4	12.698
Heterogeneous nuclear ribonucleoprotein H	HNRNPH1	2.550	3.8	5.9104
Large ribosomal subunit protein eL18	RPL18	2.495	13.8	11.018
Large ribosomal subunit protein eL30	RPL30	2.424	24.3	11.137
Superoxide dismutase [Cu‐Zn]	SOD1	2.403	15.6	6.0812
Peroxisomal multifunctional enzyme Type 2; (3R)‐hydroxyacyl‐CoA dehydrogenase; Enoyl‐CoA hydratase 2	HSD17B4	2.380	1.9	5.5537
Large ribosomal subunit protein uL6	RPL9	2.356	11.5	14.11
Periplakin	PPL	2.334	0.6	5.7
Sperm‐associated antigen 4 protein	SPAG4	2.214	2.5	6.4551
26S proteasome regulatory subunit 8	PSMC5	2.177	5.2	6.3759
Interleukin‐1 receptor antagonist protein	IL1RN	2.132	8.5	5.7813
Fructose‐2,6‐bisphosphatase TIGAR	TIGAR	2.132	4.1	5.6063
Junction plakoglobin	JUP	2.105	20.9	92.073
Proteasome subunit alpha type‐4	PSMA4	2.060	8.4	5.7268
Nuclear distribution protein nudE homologue 1	NDE1	2.029	3.9	5.8853

*Note:* Only proteins with a fold change ≥ 2 were considered upregulated. A total of 74 significantly altered proteins were identified from among 365 detected proteins using quantitative proteomics analysis.

**TABLE 2:** Downregulated proteins in pantethine (500 ng/mL)‐treated psoriatic skin equivalents compared to untreated controls.

Protein name	Protein accession	Fold change	Coverage (%) (≥ 10)	Protein score (≥ 25)
Glucose‐6‐phosphate isomerase	GPI	0.374	7.7	24.688
Keratin, Type II cytoskeletal 78	KRT78	0.295	13.7	24.502
Gasdermin‐A; Gasdermin‐A, N‐terminal; Gasdermin‐A, C‐terminal	GSDMA	0.285	23.4	37.72
Nucleoside diphosphate kinase B; Putative nucleoside diphosphate kinase	NME2	0.213	28.9	22.906
Actin, cytoplasmic 1	ACTB	0.163	67.5	34.518
Tubulin alpha‐4A chain	TUBA4A	0.119	27.5	33.915
Histone H4	H4C1	0.113	42.7	41.827
Profilin‐1	PFN1	0.091	39.3	34.59
Cystatin‐B	CSTB	0.057	45.9	57.485
Actin, alpha skeletal muscle	ACTA1	0.057	27.9	31.963
Histone H2B Type 1‐B	H2BC3 H2BC13	0.042	27.8	41.938
Collagen alpha‐2 (I) chain	COL1A2	0.030	5.6	50.913

*Note:* Only proteins with a fold change ≤ 0.5 were considered downregulated. A total of 74 significantly altered proteins were identified from among 365 detected proteins using quantitative proteomics analysis.

Upregulated proteins were predominantly associated with vesicle‐related and structural regulatory pathways. Specifically, 63.63% of upregulated proteins were classified as exosomal, including ribosomal and proteasomal components that demonstrated strong protein–protein interactions (Figure 5A). In addition to vesicle‐associated components, pantethine also upregulated proteins involved in epidermal cohesion and cytoskeletal organization, including junction plakoglobin (JUP), transgelin‐2 (TAGLN2) and keratin 6A (KRT6A).

**FIGURE 5:**
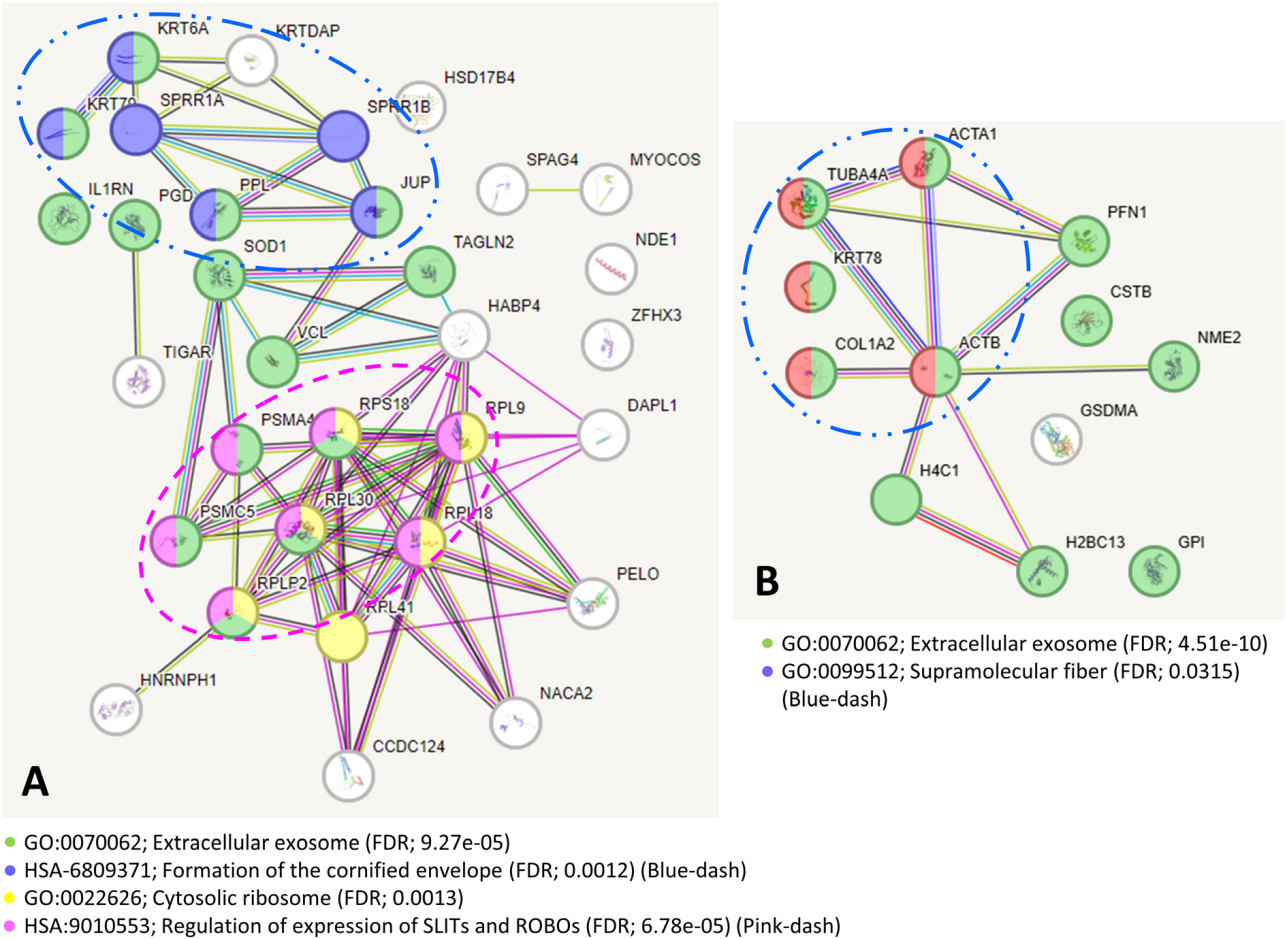
Protein interaction and classification diagram: Increased (A) and decreased (B) expression in psoriasis‐like skin following treatment with pantethine (500 ng/mL), compared to the untreated control group. Proteomic analyses were performed using three independent biological replicates per group (*n* = 3).

Network‐level analysis further indicated that upregulated ribosomal and proteasomal proteins converging on SLIT–ROBO signalling (RPL9, RPL18, RPL30, RPS18, PSMA4 and PSMC5) were among the regulated nodes, highlighting coordinated modulation of vesicle‐associated communication and intracellular regulatory pathways.

Downregulated proteins remained predominantly exosomal in origin (91.66%). These proteins included keratin family proteins (KRT78), cytoskeleton‐associated proteins (ACTB, ACTA1 and TUBA4A), and extracellular matrix components (COL1A2). STRING‐based protein interaction analysis demonstrated clustering among keratin‐, cytoskeleton‐ and vesicle‐associated proteins (Figure 5B). The resulting interaction networks represent computationally predicted functional associations derived from STRING analysis.

## 4 Discussion

This study demonstrates that pantethine modulates pathogenic cell communication and signalling networks in psoriasis using a full‐thickness 3D human skin model, while exhibiting efficacy and a favourable safety profile within a defined concentration range. By integrating histopathological, immunohistochemical, transcriptomic and proteomic analyses, our findings indicate that pantethine acts through coordinated reprogramming of disease‐associated signalling networks rather than by targeting isolated molecular effectors. This systems‐oriented analytical framework, previously established by our group [11, 26, 27, 28], provides a robust strategy for evaluating both therapeutic efficacy and underlying mechanisms in complex inflammatory skin models.

Histopathological assessment revealed a significant reduction in epidermal thickening, a cardinal feature of psoriasis, with pantethine at 500 ng/mL achieving efficacy comparable to the standard treatment Xamiol and exceeding that observed at higher (1000 ng/mL) or lower (100 ng/mL) concentrations. Consistent with these morphological improvements, immunohistochemical analyses demonstrated marked downregulation of key psoriasis‐associated proteins, including β‐defensin, caspase‐3, IL‐1β, TNF‐α and WNT, supporting a broad anti‐inflammatory effect that reflects attenuation of upstream signalling integration rather than direct cytokine blockade. Reduced caspase‐3 expression further suggests normalization of keratinocyte turnover rather than nonspecific cytotoxicity, while concomitant upregulation of Nrf‐2 and TGF‐β highlights activation of antioxidative and immunomodulatory pathways. At the transcriptional level, decreased expression of *psoriasin‐hS100A7* and related inflammatory genes, together with increased *filaggrin*, *caspase‐14* and *involucrin*, indicates partial restoration of epidermal differentiation and barrier function. Although *filaggrin* levels did not fully reach those of normal skin, their recovery within an inflammatory context is likely to be biologically meaningful.

Proteomic profiling identified 365 detected proteins, of which 74 were significantly regulated by pantethine treatment. A striking feature of this response was the coordinated suppression of vesicle‐associated proteins, suggesting that modulation of vesicle‐associated proteins may contribute to altered intercellular signalling dynamics during restoration of epidermal homeostasis. Notably, pantethine downregulated a large proportion of exosomal proteins (87.09%, 27/31), many linked to keratinocyte differentiation, cornified envelope formation and acanthosis‐related processes, consistent with a systems‐level dampening of vesicle‐mediated amplification loops. Immunohistochemical validation supported this interpretation, as CD63 expression was reduced to near‐normal levels at all pantethine concentrations, whereas CD9 suppression was prominent only at the highest dose. These dose‐dependent patterns align with lipid‐sensitive regulation of vesicle biogenesis and release and are consistent with pantethine's established effects on lipid metabolism and ceramide‐dependent signalling pathways [25, 30, 31].

Alterations in vesicle‐associated proteins occurred in parallel with broad suppression of inflammatory and proliferative signalling pathways, as reduced CD63/CD9 expression coincided with decreased β‐defensin, IL‐1β, TNF‐α and WNT levels. This convergence is consistent with reduced inflammatory activity occurring alongside alterations in vesicle‐associated proteins, although direct effects on extracellular vesicle‐mediated communication remain to be demonstrated. In parallel, increased expression of *filaggrin*, *caspase‐14* and *involucrin* reflects restoration of differentiation programs and barrier recovery, processes frequently disrupted by persistent inflammatory signalling in psoriasis [32]. Activation of Nrf‐2 together with increased TGF‐β further reinforces the concept of a coordinated anti‐inflammatory and pro‐repair response integrating vesicle suppression with redox regulation and immunomodulation.

Interestingly, pantethine did not significantly alter the expression of several pro‐inflammatory chemokines and cytokines, including CCL‐20, IL‐21, IL‐22, IL‐6 and IL‐8. This selective response pattern suggests that pantethine may not function as a broad immunosuppressive agent, but rather modulates specific inflammatory and vesicle‐associated signalling pathways linked to epidermal hyperproliferation, oxidative stress and tissue remodelling. Notably, the preserved expression of CCL‐20 together with IL‐21 and IL‐22 may indicate incomplete disruption of the adaptive immune and auto‐amplification circuitry associated with the Th17/CCR6 axis in psoriasis, despite attenuation of upstream inflammatory mediators such as IL‐17, TNF‐α and IL‐1β [33]. The persistence of these cytokine pathways may therefore reflect partial maintenance of physiological immune communication within the psoriatic microenvironment while limiting excessive inflammatory amplification.

Beyond vesicle‐associated pathways, pantethine modulated intracellular proteins critical for epidermal cohesion and cytoskeletal organization. Upregulation of JUP, TAGLN2 and KRT6A suggests enhanced structural resilience and barrier stabilization. Although JUP and TAGLN2 have not been extensively characterized in psoriasis, their known functions strongly implicate them in epidermal homeostasis and inflammatory regulation. JUP plays a key role in desmosomal adhesion and cell survival [34, 35], and its increased expression is consistent with improved epidermal cohesion. TAGLN2 contributes to actin organization, immune regulation and keratinocyte proliferation [36], potentially complementing pantethine's antioxidant and metabolic effects [37]. Mouse models of Jup deficiency display desmosome disruption, epidermal thickening and inflammation [38], reinforcing its role in maintaining epidermal homeostasis. Interestingly, KRT6A showed discordant patterns between proteomic and immunohistochemical analyses, with increased abundance detected by whole‐tissue proteomics but reduced epidermal immunostaining following pantethine treatment. Because the two approaches assess different aspects of protein abundance and spatial distribution, this discrepancy cannot be definitively resolved using the current dataset. The proteomic finding may reflect KRT6A abundance contributed by the overall tissue compartment, whereas the immunohistochemical finding specifically reflects its epidermal localization. Therefore, the KRT6A results should be interpreted cautiously and do not provide definitive evidence for either overall upregulation or suppression of KRT6A by pantethine. In contrast, downregulation of hyperproliferative and cytoskeleton‐associated proteins, including ACTB and TUBA4A, was more consistently associated with attenuation of psoriasis‐related pathological remodelling.

Pantethine also downregulated COL1A2, a structural protein commonly linked to psoriasis [39]. These proteomic findings were consistent with immunohistochemical observations showing reduced β‐defensin, IL‐1β and TNF‐α expression together with increased TGF‐β, reinforcing pantethine's profile as an anti‐inflammatory and tissue‐restorative modulator. In addition, downregulation of extracellular matrix‐associated proteins, including COL1A2, corroborated by decreased COL1A2 immunostaining, suggests partial remodelling of the chronically inflamed dermal microenvironment [39]. Pantethine additionally reduced cytoskeleton‐associated proteins, including TUBA4A [40], indicating broader effects on epidermal structural organization and vesicle‐associated cellular processes.

At the network level, proteomic interaction analysis revealed convergence of vesicle‐associated and cytosolic ribosomal proteins on regulation of the SLIT/ROBO signalling pathway (RPL9, RPL18, RPL30, RPS18, PSMA4 and PSMC5). Although individual protein changes were modest, the extremely low false discovery rate supports the robustness of this pathway‐level association. The SLIT/ROBO axis, initially characterized in axon guidance, is increasingly recognized as a regulator of immune responses, angiogenesis, and tissue organization [41], and its dysregulation has been implicated in psoriasis [42]. These findings therefore suggest a potential association between pantethine‐responsive protein networks and SLIT/ROBO‐related biological processes; however, they represent computationally inferred associations rather than direct evidence of altered SLIT/ROBO pathway activity. Reactome analysis further linked the identified ribosomal and proteasomal proteins to processes involving ROBO3 isoform regulation and protein turnover [43]. Given the absence of direct changes in canonical SLIT/ROBO pathway components or functional validation, these observations should be considered hypothesis‐generating and require experimental confirmation.

Pantethine's modulation of vesicle‐associated and structural proteins was accompanied by reduced HIF‐1α, COL1A2 and VEGF expression across proteomic and immunohistochemical datasets. These coordinated changes suggest attenuation of hypoxia‐associated inflammatory and extracellular matrix remodelling responses implicated in psoriasis progression. Potential contributing mechanisms may include Nrf‐2 and TGF‐β activation associated with reduced oxidative stress and restoration of tissue homeostasis [44], together with decreased inflammatory cytokine signalling linked to sustained hypoxia‐responsive inflammatory activity [45]. In addition, modulation of proteasomal and vesicle‐associated proteins may influence intracellular stress‐response regulation and protein turnover [46], while broader changes in cytoskeleton‐ and extracellular matrix‐associated proteins may reflect reduced tissue remodelling and inflammatory microenvironmental stress [47]. Suppression of ECM‐ and cytoskeleton‐associated proteins may further alleviate mechanical cues associated with HIF‐1–LOX–related extracellular matrix stiffness in chronic inflammatory skin conditions [48]. Collectively, these findings support an association between pantethine‐mediated modulation of inflammatory, vesicle‐associated and tissue‐remodelling responses and reduced hypoxia‐responsive signalling activity, rather than definitive regulation of a discrete SLIT/ROBO–HIF‐1α signalling axis.

Despite these insights, several limitations should be acknowledged. Although the 3D psoriatic skin model is highly disease‐relevant, it lacks systemic immune and neurovascular components that may influence pantethine's effects in vivo. In addition, the relatively limited proteomic depth obtained from full‐thickness skin lysates may have restricted detection of low‐abundance regulatory proteins and signalling pathways. The discordant KRT6A findings between whole‐tissue proteomics and epidermal immunohistochemistry could not be resolved by an independent protein‐level validation method in this study. This discrepancy highlights the importance of considering tissue compartment and spatial localization when interpreting proteomic and immunohistochemical findings. Consequently, the observed vesicle‐associated, structural and tissue‐remodelling changes should be interpreted as associative rather than definitive mechanistic interactions. Furthermore, the vesicle‐related findings in this study were inferred primarily from proteomic and immunohistochemical alterations in exosome‐associated markers rather than from direct extracellular vesicle isolation or quantitative characterization. Therefore, these observations should not be interpreted as definitive evidence of altered extracellular vesicle biogenesis or release. Similarly, the proposed involvement of SLIT/ROBO‐related signalling pathways was derived from bioinformatic pathway enrichment and protein interaction analyses and therefore requires further experimental and functional validation. Future studies incorporating optimized proteomic workflows, targeted perturbation approaches, metabolic analyses, direct extracellular vesicle characterization and in vivo or patient‐derived models will be important to further clarify pantethine's biological effects. Optimization of formulation, delivery and dosing strategies will also be essential to support translational development of pantethine as a topical or adjunctive therapeutic approach for psoriasis.

## 5 Conclusion

This study demonstrates that pantethine ameliorates psoriasis‐like epidermal pathology through coordinated modulation of inflammatory responses together with alterations in vesicle‐associated proteins, antioxidative and tissue‐remodelling responses in a full‐thickness 3D human skin model. Pantethine selectively reduced the expression of multiple pro‐inflammatory and hyperproliferative markers while promoting antioxidative and regulatory pathways associated with restoration of epidermal differentiation and barrier integrity. Proteomic and immunohistochemical analyses further identified alterations in vesicle‐associated, cytoskeletal and extracellular matrix‐related proteins, together with reduced expression of hypoxia‐associated inflammatory mediators, including HIF‐1α and VEGF. Network‐based analyses additionally identified computationally inferred associations with SLIT/ROBO‐related pathways; however, these associations remain hypothesis‐generating and require direct experimental validation. Collectively, these findings support pantethine as a promising candidate for modulation of dysregulated epidermal homeostasis and intercellular inflammatory communication in psoriasis, while highlighting the need for further functional validation of the underlying molecular mechanisms.

## Author Contributions

P.S. and S.A. conceived and designed the study, developed the methodology, curated and analysed the data, conducted the investigation, validated and visualized the results, supervised the project, acquired funding and wrote the original draft as well as revised the manuscript. P.A. contributed to study conceptualization, supervision, validation, visualization and manuscript revision. K.F. contributed to conceptualization, methodology, data curation, investigation, visualization, and manuscript drafting and revision. T.T., T.K. and W.M. contributed to methodology, investigation and manuscript revision. O.R. contributed to methodology, investigation, visualization, validation and manuscript revision. All authors read and approved the final version of the manuscript.

## Funding

This research project has been funded by Mahidol University (Fundamental Fund: fiscal year 2025 by National Science Research and Innovation Fund (NSRF)): S.A.

## Ethics Statement

The authors have nothing to report.

## Conflicts of Interest

The authors declare no conflicts of interest.

## Supporting information


**Table S1:** Reagents and materials used in this study.
**Table S2:** Primer sequences for qRT‐PCR.

## Data Availability

The proteomics dataset generated and analysed during this study has been deposited in the ProteomeXchange Consortium via the PRIDE partner repository under Accession number PXD082665 (https://doi.org/10.6019/PXD082665). The dataset will be made publicly available upon publication of the manuscript. Other data supporting the findings of this study are available from the corresponding author upon reasonable request.
