# Supplementary material for: Pantethine Restores Epidermal Homeostasis and Modulates Vesicle‐Associated Proteins in Psoriasis
Source: Exp Dermatol. 2026 Oct 30;35(11):e70381. doi: 10.1111/exd.70381 (PMC13722816; doi:10.1111/exd.70381)
Supplement: Supplementary file 1 [file EXD-35-e70381-s001.docx]

**Supplementary Table 1. Reagents and materials used in this study**

| **Category** | **Reagent / Material** | **Application** | **Supplier** | **Country** | **Catalog no.** |
| --- | --- | --- | --- | --- | --- |
| Compound | Pantethine (Coenzyme A Precursor: C_22_H_42_N_4_O_8_S_2_ 300 mg) | Treatment | NOW Foods | USA | 0487B v7 |
| Reference drug | Xamiol^®^ gel (calcipotriol 50 µg/g + betamethasone dipropionate 0.5 mg/g) | Positive control | LEO Pharma | Denmark | – |
| Cell line | HaCaT | Cytotoxicity assay | CLS Cell Lines Service | Germany | 300493 |
| 3D skin model | Psoriatic skin equivalent (SOR-300-FT) | Treatment / analysis | MatTek Corp. | USA | SOR-300-FT |
| 3D skin model | Normal skin equivalent (SOR-300-FT-CTR) | Control | MatTek Corp. | USA | SOR-300-FT-CTR |
| Viability assay | MTT reagent | Cytotoxicity | Sigma-Aldrich | USA | – |
| IHC antibody | β-defensin | Immunohistochemistry | MyBioSource | USA | MBS1490249 |
| IHC antibody | TNF-α | Immunohistochemistry | MyBioSource | USA | MBS820357 |
| IHC antibody | IL-1β | Immunohistochemistry | MyBioSource | USA | MBS127158 |
| IHC antibody | IL-6 | Immunohistochemistry | MyBioSource | USA | MBS2001862 |
| IHC antibody | IL-8 | Immunohistochemistry | MyBioSource | USA | MBS9607651 |
| IHC antibody | IL-17 | Immunohistochemistry | MyBioSource | USA | MBS2032548 |
| IHC antibody | IL-21 | Immunohistochemistry | MyBioSource | USA | MBS8247452 |
| IHC antibody | IL-22 | Immunohistochemistry | MyBioSource | USA | MBS8305903 |
| IHC antibody | CCL-20 | Immunohistochemistry | MyBioSource | USA | MBS631195 |
| IHC antibody | TGF-β | Immunohistochemistry | MyBioSource | USA | MBS8242488 |
| IHC antibody | Nrf-2 | Immunohistochemistry | MyBioSource | USA | MBS9608128 |
| IHC antibody | Caspase-3 | Immunohistochemistry | MyBioSource | USA | MBS9700614 |
| IHC antibody | WNT | Immunohistochemistry | MyBioSource | USA | MBS1497402 |
| IHC antibody | CD63 | Immunohistochemistry | MyBioSource | USA | MBS8292185 |
| IHC antibody | CD9 | Immunohistochemistry | MyBioSource | USA | MBS9607221 |
| IHC antibody | KRT6A | Immunohistochemistry | MyBioSource | USA | MBS2070742 |
| IHC antibody | COL1A2 | Immunohistochemistry | MyBioSource | USA | MBS220762 |
| IHC antibody | HIF-1α | Immunohistochemistry | MyBioSource | USA | MBS9203233 |
| IHC antibody | VEGF | Immunohistochemistry | MyBioSource | USA | MBS140026 |
| RNA extraction kit | RNeasy Mini Kit | qRT-PCR | Qiagen | Canada | – |
| qPCR reagent | iTaq SYBR Green Supermix | qRT-PCR | Bio-Rad | USA | – |
| Proteomics reagent | RapidGest SF | Sample prep | Waters | USA | – |
| Proteomics reagent | Trypsin (sequencing grade) | Digestion | Promega | USA | – |

**Supplementary Table 2. Primer sequences for qRT-PCR**

| **Gene** | **Forward** | **Reverse** |
| --- | --- | --- |
| *FLG* | 5′-AAGGTTCACATTTATTGCCAAA-3′ | 5′-GGATTTGCCGAAATTCCTTT-3′ |
| *β-Actin* | 5′-TGACGGGGTCACCCACACTGTGCCCATCTA-3′ | 5′-CTAGAAGCATTTGCGGTGGACGATGGAGGG-3′ |
| *Caspase-14* | 5′-AAATGAGCAATCCGCGGTCTTTGG-3′ | 5′-CCGTGGAATAAACGTGCAAGGCAT-3′ |
| *Involucrin* | 5′-CTCCACCAAAGCCTCTGC-3′ | 5′-CTGCTTAAGCTGCT GCTC-3′ |
| *Psoriasin-hS100A7* | 5′-AGACGTGATGACAAGATTGAC-3′ | 5′-TGTCCTTTTTCTCAAAGACGTC-3′ |
| *Koebnerisin 15S-hS100A15S* | 5′-CAAGTTCCTTCTGCTCCATCTTAG-3′ | 5′-AGCCTTCAGGAAATAAAGACAATC-3′ |
| *Koebnerisin 15L-hS100A15L* | 5′-ACGTCACTCCTGTCTCTCTTTGCT-3′ | 5′-TGATGAATCAACCCATTTCCTGGG-3′ |
